# #EnufSnuff.TXT-FirstResponder: a pilot randomized controlled trial of a text message intervention for smokeless tobacco cessation among First Responders

**DOI:** 10.3389/fpubh.2025.1699800

**Published:** 2026-01-19

**Authors:** Devon Noonan, Laura J. Fish, Susan G. Silva, Mariana Da Costa, Leigh Ann Simmons, Norma Garcia Ortiz, Courtney Swinkels, Daum Jung, Herbert H. Severson, Kathryn I. Pollak

**Affiliations:** 1 Duke University School of Nursing, Durham, NC, United States; 2 Duke Cancer Institute, Durham, NC, United States; 3 Department of Family Medicine and Community Health, Duke University, Durham, NC, United States; 4 College of Health and Human Sciences, Western Carolina University, Cullowhee, NC, United States; 5 University of California Davis Betty Irene Moore School of Nursing, Sacramento, CA, United States; 6 Oregon Research Institute, Eugene, OR, United States; 7 Department of Population Health Sciences, Duke University, Durham, NC, United States

**Keywords:** cessation intervention, first responders, m-Health, smokeless tobacco, tobacco cessation

## Abstract

**Introduction:**

First Responders are three times more likely to use smokeless tobacco than those in the general population and very few targeted cessation interventions are available.

**Methods:**

The #EnufSnuff.TXT First Responder text-based intervention (*n* = 30), which includes an optional reduction component and tailored text-messages for cessation, was administered alongside the Enough Snuff Intervention (*n* = 30), which includes a cessation booklet and general support texts. Both groups received a 4-week supply of 4 mg Nicotine Replacement Therapy (NRT) lozenges to assist with cessation. We surveyed participants 30 days post-intervention initiation to assess feasibility, acceptability, and Smokeless Tobacco cessation outcomes. A subset of participants participated in qualitative interviews to assess feedback on the intervention.

**Discussion/conclusion:**

Feasibility benchmarks for recruitment, retention, and engagement were met. Both programs helped First Responders quit. The respective quit rate for #EnufSnuff.TXT-FR arm was higher compared to Enough Snuff was 40% *vs* 33% (OR = 1.33, *p* = 0.5925; RR = 1.20, *p* = 0.5935) for the intent-to-treat cases and 52% *vs* 44% (OR = 1.41, *p* = 0.5555; RR = 1.20, *p* = 0.5570) for the completers. A text-based cessation intervention was found to be feasible and represents a scalable intervention approach and both interventions produced high quit rates. Larger scale efficacy testing is warranted.

**Clinical trial registration:**

https://clinicaltrials.gov/study/NCT05111041?term=NCT05111041&rank=1, identifier NCT05111041.

## Introduction

First Responders (e.g., firefighters, emergency medical technicians and paramedics) are a high-risk group for chronic disease given high rates of tobacco use (1–3). Firefighters and EMTs represent a high-risk group for cancer given the occupational exposures associated with extinguishing fires and responding to other emergencies (3, 4). Because First Responders already have these unmodifiable risk factors, there is a critical need to identify and eliminate any other modifiable risk factors of cancer, such as smokeless tobacco (SLT) use. SLT use, (chewing tobacco, snuff, snus, etc.) is high among firefighters with 10–18% using compared to 3% in the general population (1, 5). Using SLT increases the risk of developing head and neck cancers; the prevalence of head and neck cancer among U.S. firefighters exceeds that in the general population (6). Further, the late initiation rate (e.g., starting use of SLT after joining the fire service) is as high as 16%, which is significantly higher than the population initiation rate (0.8%) and comparable to other high-risk groups like the military (14%) (1). These high late initiation rates suggest that the occupational culture of firefighters may promote SLT use (1, 5–7). Although many firefighter service organizations have supported the profession becoming tobacco-free, most policies have focused solely on cigarette smoking. This exclusion of focus on SLT might have inadvertently cultivated a growing acceptance of SLT as part of the firefighter culture (7, 8).

Given the unique contextual and occupational reasons for SLT use among firefighters and paramedics, including job stress from dangerous work conditions, exposure to traumatic events, exposure to heat and smoke (firefighters), emergency care (paramedics), sleep deprivation, the pressure to stay physically fit, and high levels of cancer fatalism, targeted interventions are needed to address SLT (1, 9). Currently, very few SLT cessation interventions exist that are personalized to the needs of First Responders. Formative research in this population highlights the unique factors that influence cessation including internal (i.e., health effects) and external (i.e., family and friends and cost) motivators for quitting that may increase the salience of cessation interventions (10). Jitnarin et al. (11) recently beta tested QUIT SPIT, a culturally tailored version of the 54-page adaption of the Enough Snuff booklet specific for firefighters and found the intervention to be feasible and acceptable among those who wanted to quit. Firefighters indicated that the intervention increased their confidence in the ability to quit. They also indicated that the delivery of the messages via an M-health platform (app or website) may be preferable to the print version, due to the flexibility and ease that it could provide while working at an occupation with varying shifts. M-health interventions for tobacco cessation have demonstrated efficacy for improving tobacco cessation and have great potential to facilitate participant engagement and activation by reaching people in a real-world setting (12–16). Previous research indicates that features that promote engagement (e.g., interactive text messaging) are positively associated with quit rates (17). Although there has been less M-health research for smokeless tobacco cessation compared to cigarette cessation, the few studies available suggest it is efficacious (18, 19). To meet this need, we adapted the #EnufSnuff.TXT-FirstResponder (FR) from our original #EnufSnuff.TXT intervention that included a text-based scheduled gradual reduction component paired with support messages to promote cessation (19, 20). We adapted text message content based on themes from interviews with 19 First Responders, including firefighters, EMTs, and paramedics (mirroring our targeted subject population), and integrated this into the intervention. In this pilot study, we aimed to test the feasibility, acceptability and preliminary efficacy of the #EnufSnuff.TXT-FR intervention compared to a widely available SLT intervention, the Enough Snuff intervention, which included a cessation booklet with a separate library of non-targeted cessation texts.

## Materials and methods

### Design

This study was a pilot randomized controlled trial comparing the #EnufSnuff.TXT-FR intervention (experimental, *N* = 30) to the Enough Snuff intervention (control, *N* = 30) on SLT use cessation. We recruited participants from our collaborating Central North Carolina Fire Department connections and social media (Facebook) ads that extended across North Carolina, South Carolina, Virginia and the broader southeast region. We screened participants from July through September 2023. We consented those deemed eligible electronically and then randomized them to either the #EnufSnuff.TXT-FR intervention or Enough Snuff intervention arm using a 1:1 treatment allocation ratio and a permuted block randomization scheme. We emailed participants their surveys at baseline and 30-days post-10-week intervention completion. We conducted post-intervention interviews with a voluntary subset of participants (*N* = 12) to obtain feedback on the intervention.

### Sample

We recruited First Responders who use SLT via social media and through flyers at local fire stations. For social media recruitment, we linked participants to a study landing page where they could sign up and be contacted about study participation. We screened interested participants for eligibility via REDCap (21, 22) including being 18 years of age or older, English-speaking, having used SLT regularly for the last year, working as either an employed (professional) or volunteer first responder (firefighter or paramedic), interested in participating in the program, willing to refrain from using any other tobacco product (such as e-cigarettes and cigarettes) for the length of the study, and having access to a cell phone with unlimited texting and good service.

### Procedure

After obtaining approval from Duke University Health System Institutional Review Board for Clinical Investigations (DUHS IRB) under protocol Pro00109404, we consented eligible participants, who were then given the baseline assessment, and randomized remotely via REDCap. Participants were contacted via email to complete follow-up surveys at 30-day post-intervention. We gave participants the option to participate in a phone interview after survey completion with a member of the study team. Participants were only compensated for completion of surveys—$15 for the baseline survey and $20 for the 30-day post-intervention survey. To ensure research transparency and rigor, the study was registered with ClinTrials.gov under NCT05111041.

### Intervention

#### Development phase

We adapted the original #EnufSnuff.TXT intervention (19, 20) to incorporate feedback from First Responders to develop the #EnufSnuff.TXT-FR intervention for this pilot. We conducted 19 remote individual interviews with First Responders including firefighters, EMTs, and paramedics. We gathered feedback on unique attributes of SLT use related to occupational factors and identified their needs and wants for a M-health cessation intervention. For example, we asked participants to provide feedback on how shift work contributed to their smokeless use habits and how this type of work may affect intervention engagement. We identified salient benefits and barriers to quitting for First Responders. We also presented trigger materials, which included the content of messages of our previous text-based SLT cessation intervention to adapt previously successful messaging to the culture and context of SLT use among First Responders.

The data was analyzed using a rapid qualitative approach (23–26). We developed a deductive template based on domains that guided the development of the original intervention including Social Cognitive Theory and the Health Belief Model (27). Our team tested and evaluated the standard template by all coding one interview and compared and resolve discrepancies. The template was revised based on the initial coding. All transcripts were double coded and the team met to discuss and resolve discrepancies. Individual summaries were combined into a matrix and domains were summarized across participants.

We used the findings from the qualitative analysis to adapt the text messages from the original #EnufSnuff.TXT program to address the unique attitudes and behaviors reported by first responders. Our formative qualitative work indicated that first responders needed additional real-time support features, including the text keyword of CALL. First Responders could text this keyword if they were experiencing work-related stress from responding to an emergent call that triggered tobacco cravings. These messages focused around providing mindfulness and deep breathing activities to deescalate triggers, such as stress, after responding to a call. We also learned that some first responders preferred a reduction to quit approach, however, the scheduled reduction text program we had tested in EnufSnuff would not work with the job demands for First Responders. Thus, we determined that we would offer participants a choice between abrupt and gradual reduction to quit, The reduction to quit approach would provide target number of dips per day that decreased to 0 over 10 weeks. We also learned that most wanted autonomy and a quit method that worked for them and NRT was commonly mentioned to support cessation.

#### Intervention arms

While we randomized participants into two different intervention arms, we did provide participants in both arms a 4-week supply of 4 mg nicotine lozenge with instructions on proper use.

##### #EnufSnuff.TXT first responder

The #EnufSnuff.TXT-FR intervention consisted of a text program that supported either abrupt cessation or reduction to quit. We assessed participant quitting preferences (e.g., abrupt vs. reduction cessation approach) on day 1 of the intervention. Based on their response, we placed them into their preferred approach. This intervention included 10 weeks of support text messages, real-time support via interactive keyword text messages, and NRT.

*Reduction cessation preference*: We based reduction on participant’s baseline dips/chews per day and reduced over the 6-week reduction period. We sent participants a text at the beginning of each week alerting them to their number of dips for each day that week. We sent them a message reminder in the morning: “Do your best to only dip 6 times today, limit dip in your mouth to 30 min.” At the end of each day, we asked them to report how many times they dipped that day. If participants went more than 36 h without reporting their daily dip total, they were sent a reminder text. On their quit date, we texted participants that they had reached their quit date as well as a reminder to begin using their NRT to help curb their cravings.

*Abrupt cessation preference*: We asked participants to set a quit date within 2–4 weeks. We sent reminder messages leading up to their quit date. On their quit date, we texted participants that they had reached their quit date as well as a reminder to begin using their NRT to help curb their cravings.

*Support messages:* Participants received 10 weeks of support messages. These messages focused on the unique first responder barriers to cessation. We sent messages daily (range of 1–3 messages per day) and focused on general cessation content including increasing motivation and self-efficacy to quit, perceived risk of use, perceived benefits of quitting, as well as managing withdrawal, triggers, and cravings for SLT use. We also included specific messaging on the occupational health risks associated with tobacco use and job-related stress reduction strategies.

*Real-time support:* Participants could text key words (e.g., CRAVE, SLIP, CALL, etc.) to receive real-time automated support during the program to address cravings, stress reduction, work-related triggers, and mindfulness strategies to support potential slips. Messages were all sent automatically by the texting platform Mosio (28). Study staff monitored the platform during the study.

##### Enough snuff

*Abrupt verse reduction quitting preference:* As we did on Day 1 in the intervention group, we asked those receiving the Enough snuff booklet if they preferred to quit via reduction or abrupt methods. The intervention did not change based on this preference as the Enough Snuff Cessation Booklet contains information on both abrupt and reduction cessation methods.

*Booklet*: We mailed the Enough Snuff Cessation Booklet (29), developed by Dr. Herb Severson, support texts that mapped to book content as well as NRT. The booklet is a self-help cessation intervention endorsed by the NCI in their Research-Tested Intervention Programs (RTIPs). The Enough Snuff Program has been shown to be efficacious in promoting SLT cessation in the general population with quit rates ranging from 16 to 18% (30, 31). To deliver the most equivalent intervention to the original Enough Snuff Program, we sent participants motivational text messages that map to the content of the booklet. One week after the cessation booklet was sent, we texted participants twice a week for the next 10 weeks. These are separate texts than what the intervention group received and coincided with the Enough Snuff Booklet. The messages focused on: motivation to quit, setting a quit date, picking a quit plan, motivation to follow-through with plan and dealing with tough situations when quitting. Messages referred to the Enough Snuff Booklet and asked for a response from participants. We chose to send text messages to participants instead of phone calls as texting is consistent with our mode of contact with participants, more cost-effective and scalable, expands our reach, and is highly acceptable according to our pilot work.

#### Measures

##### Feasibility

Feasibility outcomes were: (a) the number of people who use SLT recruited and randomized during the 4-month recruitment period; and (b) the retention rates in the experimental arm compared to the control arm at 30-day post-intervention, as measured by number and percent of those randomized who completed the 30-day post-intervention survey. *A priori* benchmarks were: (a) ability to recruit and enroll 50 participants in 4-months; and (b) ability to retain 80% of the sample at the 30-day post-intervention follow-up. Additionally, feasibility outcomes included two measures of intervention engagement as measured by the proportion of messages read per participant. To calculate the proportion of messages read per participant, we used a 5-point Likert scale to ask participants the following two questions: “What did you typically do when you received messages??” (1 = ignored it completely, 5 = read it right away), “On a typical day, did you read messages you received?” (1 = Not at all, 5 = read the whole message).

##### Acceptability

We determined acceptability based on participants’ ratings of (a) the usefulness of the intervention received (Not at all useful = 1, 5 = extremely useful); and (b) whether they would recommend the program to a friend (Not likely to recommend = 1, 5 = extremely likely to recommend).

##### Preliminary efficacy

We assessed preliminary efficacy of the intervention using self-report 7-day point prevalence abstinence (quit rate) at 30-day post-intervention. The median percent change in number of chews per day per participant during the past 7 days was assessed as a secondary outcome for those that did not report quitting at 30 days post-intervention.

### Data analysis

We used descriptive statistics to detail the sample characteristics and outcomes. Non-directional statistical tests were performed with significance set at 0.05 per test.

#### Sample characteristics

We tested intervention differences in baseline characteristics using Wilcoxon Two-Sample Tests for continuous measures and Fisher’s Exact tests for categorical measures due to the small sample size per arm.

#### Feasibility and acceptability outcomes

We dichotomized feasibility and acceptability outcomes score of 4 or 5 vs. score of 1–3. For each dichotomized outcome, we compared between-intervention differences in the proportions using Fisher’s Exact tests.

#### Preliminary efficacy

The outcome was SLT cessation (quit) rate per intervention arm at 30-days. In intention-to-treat (ITT) analysis, we included participants randomized to a treatment arm regardless of study or treatment completion. For the ITT analysis, those who did not complete the 30-day post-intervention assessment were considered to have continued to use SLT. In completers analysis, we included those who completed the 30-days post-intervention assessment. The efficacy analysis focused on effect size due to the exploratory nature of this small pilot study. We used logistic regression to test for intervention differences in SLT cessation. We used both odds ratios (OR) and relative risk (RR) along with their 95% confidence intervals (CI) to estimate effect sizes and address clinical significance. Among the subset of completers who continued to use SLT, a non-parametric Wilcoxon Two-Sample Test was used to compare intervention effects on percent reduction of dips/chews per day. This subgroup analysis was primarily descriptive in nature due the very small sample sizes compared.

#### Statistical power

Past SLT cessation studies (both text and web-based) have yielded ITT quit rates in the 12–20% range. We estimated *a priori* that the #EnufSnuff.TXT-FR and Enough Snuff arms would yield 15 and 10% quit rates, respectively. Assuming a small effect size (OR = 1.5), a sample size of 60 (30/arm) did not provide 80% power to detect a statistically significant difference in quit rates with significance set at 0.05. Thus, the preliminary efficacy analyses for this small pilot study focused on estimating effect sizes and their CIs.

#### Qualitative methods

We used directed content analysis to explore existing theories to analyze interview data (32). This type of content analysis allows for a broad collection of thoughts and opinions, and therefore, was fitting given our goal of collecting data to improve programmatic components of the intervention (33). We conducted data analysis to systematically identify and organize themes. We then identified subthemes for broad categories through a visual inspection of the data.

## Results

### Sample characteristics

See Table 1 for sample characteristics. We randomized a total of 60 First Responders, with 30 randomized to receive the #EnufSnuff.TXT-FR intervention and 30 assigned to the Enough Snuff intervention. Most participants reported being firefighters (65%), followed by paramedics (25%). Most (85%) were non-volunteer professionals. All identified as male, except for one female participant. Age ranged from 20 to 62 years, age started using chew/snuff regularly ranged from 10 to 33 years, and years of SLT use ranged from 1 to 41 years. All 60 participants reported using chew/snuff at least 4 times per week, with 57 (95%) indicating using 7 days per week. The median times per day reported using chew/snuff was 10.0 (range: 3–30), and 82% used 3 or more cans/pouches of chew/snuff per week. The two intervention groups did not significantly differ on any sample characteristics. The 60 participants were recruited from seven states, with most from North Carolina (45%, *n* = 27), followed by South Carolina (27%, *n* = 16), and Virginia (22%, *n* = 13). Florida, Georgia, Tennessee, and Texas had one participant each (1.7% each).

**Table 1:** Sample characteristics.

Baseline characteristic	Total *N* = 60 *n* (%)	#EnufSnuff.TXT-FR *N* = 30 *n* (%)	Enough Snuff *N* = 30 *n* (%)	*p*-value
First responder category				0.7975
Firefighter	39 (65%)	18 (60%)	21 (70%)	
Paramedic	15 (25%)	8 (27%)	7 (23%)	
EMT	3 (5%)	2 (7%)	1 (3%)	
Police	3 (5%)	2 (7%)	1 (3%)	
First responder status				0.4716
Professional	51 (85%)	27 (90%)	24 (80%)	
Volunteer	9 (15%)	3 (10%)	6 (20%)	
Demographics & SLT history				
Male gender	59 (98%)	29 (97%)	30 (100%)	—
Post-secondary education	47 (78%)	23 (77%)	24 (80%)	0.7540
White race	57 (95%)	28 (93%)	29 (97%)	1.0000
Hispanic/Latinx	1 (2%)	0 (0%)	1 (3%)	—
Difficulty paying bills	1 (2%)	0 (0%)	1 (3%)	—
Married/living with partner	48 (80%)	25 (83%)	23 (77%)	0.5186
Number cans/pouches of chew/snuff used per week				1.0000
Less than 2 cans/pouches	3 (5%)	2 (7%)	1 (3%)	
2–3 cans/pouches	8 (13%)	4 (13%)	4 (13%)	
3 or more cans/pouches	49 (82%)	24 (80%)	25 (83%)	

Demographics and SLT history	Median (25th, 75th)	Median (25th, 75th)	Median (25th, 75th)	*p*-value
Age, in years	35.0 (30.0, 44.5)	35.5 (29.0, 44.0)	34.0 (31.0, 45.0)	0.9587
Age started using chew/snuff regularly	16.0 (15.0, 19.0)	17.0 (15.0, 19.0)	16.0 (15.0, 19.0)	0.8697
Years of SLT use	19.0 (13.0, 25.5)	18.5 (13.0, 24.0)	19.0 (13.0, 28.0)	1.0000
Number times use chew/snuff per day	10.0 (7.0, 13.5)	10.0 (7.0, 15.0)	9.0 (7.0, 10.0)	0.1492

SLT = smokeless tobacco use; Median (25th, 75th) reported due to small sample sizes and/or skewness. (—) indicates no *p*-value for intervention arm comparison due to low frequency in total sample. Fisher’s Exact Tests for categorical characteristics and Wilcoxon Two-Sample Tests for continuous measures.

### Feasibility

**Figure 1:**
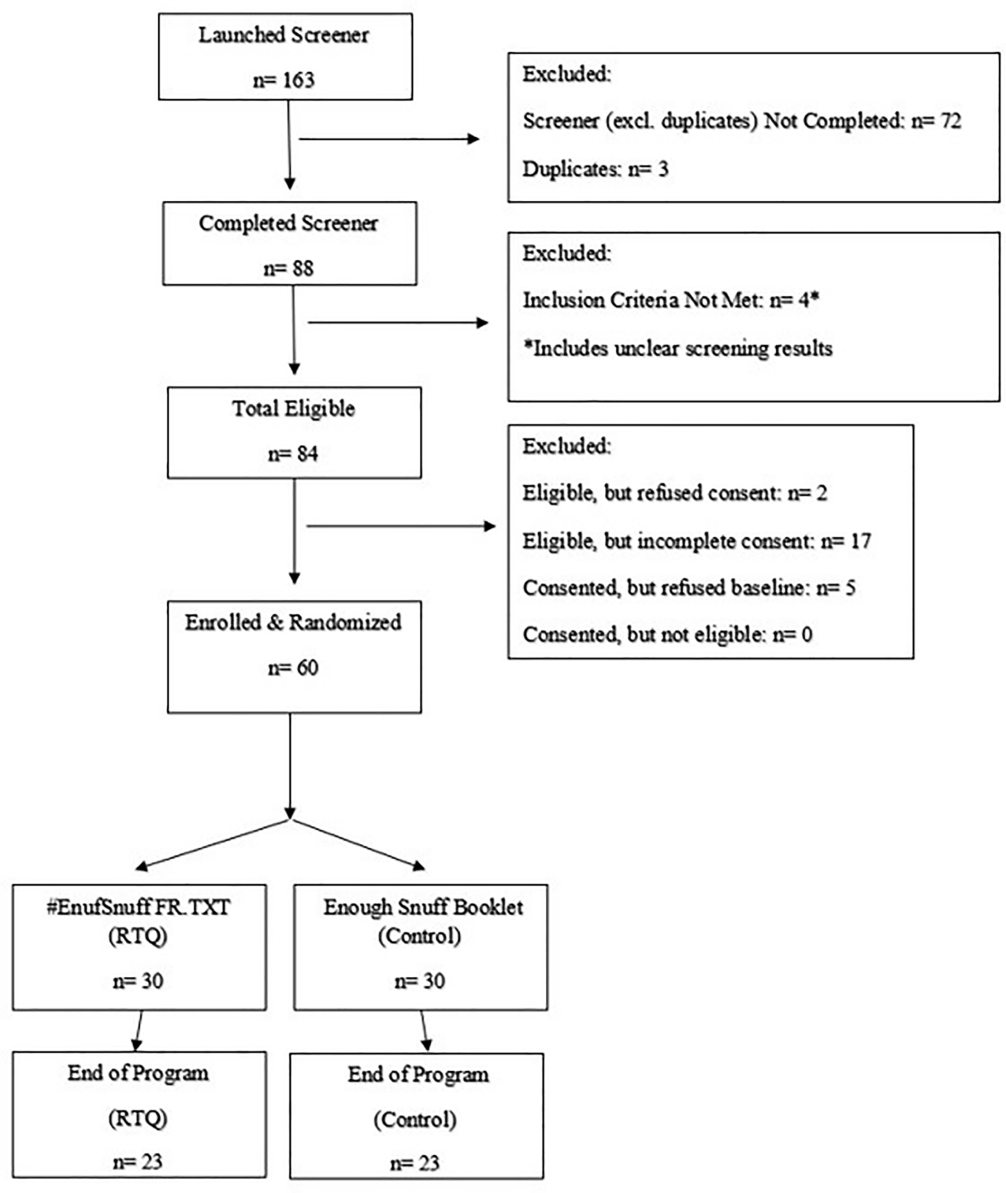
Consort diagram.

Staff screened 163 individuals. Of these, 60 (37%) were subsequently enrolled and randomized to an intervention arm. Thus, the study exceeded our benchmark of being able to recruit and enroll 50 First Responders in 4 months. Among the 60 randomized, the retention rate was 77% at 30-day post-intervention follow-up for the total sample (*n* = 46) and per intervention arm (*n* = 23/arm) (See Figure 1 Consort Diagram). There were no significant sample characteristic differences among those participating in the 30-day follow-up (*n* = 46) compared to those who did not (*n* = 14). Table 2 shows the responses to the feasibility (engagement) and acceptability questions. A total of 36 of the 60 participants (60%) completed these items. Among the 36 providing feedback, 89% reported typically reading messages when they had time or right away, and 89% reported reading 75% or more of a message. The intervention arms did not significantly differ on engagement (*p* > 0.05).

**Table 2:** Feasibility and acceptability.

Feasibility: engagement	Total *N* = 36 *n* (%)	#EnufSnuff.TXT-FR *N* = 19 *n* (%)	Enough Snuff *N* = 17 *n* (%)	*p*-value
What did you typically do when you received the messages?				
(1) Ignore it completely	1 (2.8%)	1 (5.3%)	0 (0.0%)	
(2) Read it occasionally	3 (8.3%)	2 (10.5%)	1 (5.9%)	
(3) Read it when it had time	16 (44.4%)	4 (21.1%)	12 (70.6%)	
(4) Read it right away	16 (44.4%)	12 (63.2%)	4 (23.5%)	
Read it when had time/right away	32 (88.9%)	16 (84.2%)	16 (94.1%)	0.6052
On a typical day, did you read messages you received?				
(1) Not at all	1 (2.8%)	1 (5.3%)	0 (0.0%)	
(2) Read about a 25% of the message	2 (5.6%)	1 (5.3%)	1 (5.9%)	
(3) Read about 50% of the message	1 (2.8%)	0 (0.0%)	1 (5.9%)	
(4) Read about 75% of the message	3 (8.3%)	0 (0.0%)	3 (17.7%)	
(5) Read the whole message	29 (80.6%)	17 (89.5%)	12 (70.6%)	
Read 75% or more of a message	32 (88.9%)	17 (89.5%)	15 (88.2%)	1.0000
Acceptability	*N* = 36	*N* = 19	*N* = 17	
How useful was the intervention in helping you quit chew/dip?				
(1) Not at all useful	6 (16.7%)	4 (21.1%)	2 (11.8%)	
(2) Somewhat useful	10 (27.8%)	7 (36.8%)	3 (17.7%)	
(3) Undecided	9 (25.0%)	3 (15.8%)	6 (35.3%)	
(4) Very useful	10 (27.8%)	5 (26.3%)	5 (29.4%)	
(5) Extremely useful	1 (2.8%)	0 (0.0%)	1 (5.9%)	
Very/extremely useful	11 (30.6%)	5 (26.3%)	6 (35.3%)	0.7206
Would you recommend the program to a friend?				
(1) Not at all	2 (5.7%)	1 (5.3%)	1 (6.3%)	
(2) Somewhat	3 (8.6%)	2 (10.5%)	1 (6.3%)	
(3) Undecided	8 (22.9%)	5 (26.3%)	3 (18.8%)	
(4) Very much	12 (34.3%)	5 (26.3%)	7 (43.8%)	
(5) Extremely	10 (28.6%)	6 (31.6%)	4 (25.0%)	
Very much/extremely	22 (62.9%)	11 (57.9%)	11 (68.8%)	0.7267

*N*, Data Available; Fisher’s Exact test *p*-values for dichotomized outcomes.

### Acceptability

Among the 36 who completed the acceptability questions, 58% reported the intervention was “very/extremely” or “somewhat” useful. In addition, 63% indicated that they would “very much/extremely” recommend the intervention to a friend. The interventions did not significantly differ on the two measures of acceptability.

**Table 3:** Quit rates at the 30-day post-intervention follow-up.

Analysis	Total *n* (%)	#EnufSnuff.TXT-FR *n* (%)	Enough Snuff *n* (%)	Odds Ratio (95% CI)	*p*-value
Intent-to-treat	*N* = 60	*N* = 30	*N* = 30		
Quit	22 (36.7%)	12 (40.0%)	10 (33.3%)	1.33 (0.47–3.82)	0.5925
Completers	*N* = 46	*N* = 23	*N* = 23		
Quit	22 (47.8%)	12 (52.2%)	10 (43.5%)	1.41 (0.44–4.53)	0.5555

Quit = Cessation of Smokeless Tobacco (SLT) use, based on dips/chew in the past 7 days; For the Intent-to-treat (ITT) analysis, missing data set to “0, no quit”; Logistic regression analysis.

### Real-time support messages

Among those in the #EnufSnuff.TXT-FR arm, real-time support use was minimal. Craving management support: no message (87%, *n* = 26), 1 message (10%, *n* = 3), and 3 messages (3%, *n* = 1). SLIP support message after quitting: no message (93%, *n* = 28) and 2 messages (7%, *n* = 2). Stress support: no message (93%, *n* = 28) and 2 messages (7%, *n* = 2). Call support if they needed a message after taking a work-related call: no message (97%, *n* = 29) and 1 message (3%, *n* = 1).

### Quit method preferences

At baseline, 59 of the First Responders indicated their preferred method to quit. Of those, 44 (75%) preferred a reduction approach to quit and 15 (25%) preferred an abrupt cessation method. The intervention groups did not significantly differ in the preferred method (*p* = 0.1161). Among the 29 in the #EnufSnuff.TXT-FR arm, 19 (66%) preferred a reduction approach and 10 (34%) preferred an abrupt method. Among the 30 in the Enough Snuff arm, 25 (83%) preferred a reduction approach and 5 (17%) preferred an abrupt method.

### Preliminary efficacy

The #EnufSnuff.TXT-FR arm had a higher quit rate at the 30-day post-intervention follow-up relative to the Enough Snuff arm for both the ITT and completers analyses (Table 3). The respective quit rate for #EnufSnuff.TXT-FR arm compared to Enough Snuff was 40% *vs* 33% (OR = 1.33, *p* = 0.5925) for the intent-to-treat cases and 52% *vs* 44% (OR = 1.41, *p* = 0.5555) for the completers, indicating small intervention effects (Table 4). Since this is an exploratory RCT, the relative risk for having a positive outcome (quit) in the #EnufSnuff.TXT-FR arm relative to Enough Snuff was estimated. The RR of quitting was 1.20 for both the intent-to-treat analysis (95% CI = 0.61–2.34, *p* = 0.5935) and completers (95% CI = 0.65–2.20, *p* = 0.5570). Among the 24 who completed the 30-day post-intervention assessment and reported continued use of SLT, there was no intervention difference in percent reduction in dips/chews per day per participant. The median percent reduction per participant was 50% for #EnufSnuff.TXT-FR compared to 57% for Enough Snuff (Wilcoxon Two-Sample Test, *p* = 0.7710, Table 4).

**Table 4:** Percent reduction in dips/chews per day among completers who did NOT quit.

Completers’ analysis: those continued to use SLT	Total *N* = 24	#EnufSnuff.TXT-FR *N* = 11	Enough Snuff *N* = 13	*p*-value
Percent reduction				0.7710
Median	50.0	50.0	57.1	
25th, 75th percentile	40.0, 71.7	40.0, 70.0	40.0, 73.3	
Minimum, maximum	0.0, 85.0	0.0, 85.0	0.0, 80.0	

Wilcoxon Two-Sample Test.

### Qualitative interviews

Common themes from the post-intervention interviews included that the number of messages was overwhelming at times. Many participants noted that they just stopped reading some of the messages and that they became “white noise” in the background after a while. Although others liked having multiple messages per day as it allowed them to “pause” and think before using SLT. Many participants also wanted more interaction as part of the intervention, with participants noting that the intervention at times felt “transactional” at times verse interactive. In terms of implementation, some suggested that integrating this intervention as part of health classes or other trainings would be useful. Positive feedback included that the reflection and goal-setting aspects of the program were helpful and that participants could easily rely on the scheduled delivery of the texts. Providing free NRT was viewed positively by many, although some had mixed feelings on whether it helped control their cravings.

## Discussion

This is one of the first studies to examine the feasibility and acceptability of a text-based SLT intervention for First Responders. We found high quit rates in both intervention arms. We also found that our #EnufSnuff.TXT-FR intervention produced higher quit rates in the short term compared to the widely available Enough Snuff Intervention. We met feasibility benchmarks for recruitment, retention, and engagement suggesting that our intervention is highly feasible in this population.

We did not meet our acceptability benchmarks, which differs from what we have seen in some of our prior trials (19, 20). Our qualitative interview data highlight findings that may inform these lower acceptability levels. First, participants thought the number of texts was overwhelming, suggesting that we need to reduce the number of daily texts participants receive. The maximum number of texts received per day was 5, and it appears that this may be too high for some. Participants appreciated autonomy in the cessation process, so future iterations of this intervention asking participants for the best time for messaging and preferred amount may be beneficial. This concept of autonomy in cessation and how much autonomy lends to successful cessation should also be further explored in future trials. Participants also reported wanting more interactive content included in the intervention. Although we had included multiple opportunities for interaction through use of our keywords, these keywords for real time messaging were minimally used by participants. This suggests that we need to better integrate these keywords into the intervention messaging so participants are aware they can use them. We also need to consider more integration of interactive components into our intervention in the future, such as cost calculators and messages that prompt responses and reflection. Despite these challenges, there are multiple positive intervention aspects that also emerged from both our formative and summative qualitative work with this population including access to NRT and the autonomy in choosing to quit either by reduction or abruptly that bolsters the potential acceptability of the intervention and warrants further exploration to inform future cessation trials.

Although our quit rates were high in both our intervention arms in this study, we had higher quit rates in the #EnufSnuff.TXT-FR group, albeit they were not statistically significant. Our quit rates in this study were higher than those reported for a similar text-based cessation interventions (19). One reason for this could be the addition of NRT as part of this intervention. In our formative work with First Responders, we heard that most wanted NRT included as part of the intervention. Although the evidence is mixed on the efficacy of NRT for SLT cessation (34), providing NRT for SLT cessation is typically seen at the local level (health clinics/departments, quit lines, state-level tobacco cessation programs) and should be tested as an adjunct to SLT interventions in the future, especially in populations with high rates of use and low quit rates. Understanding more about NRT preferences and reasons for use (including higher dependence levels, health considerations, economic factors) should be explored in future studies. The median percent reduction was greater in the Enough Snuff group compared to our #EnufSnuff.TXT-FR. This is a similar finding to what we saw in our original #EnufSnuff.TXT trial (19). This finding is not surprising given both interventions provided support for reduction-based quitting methods.

Finally, most participants preferred quitting using reduction methods versus abrupt quitting. This is an interesting finding that can inform future SLT cessation interventions. Although abrupt quitting is widely recommended for people who smoke cigarettes, there is little guidance for people who use SLT. The evidence for cigarette smoking on abrupt versus gradual cessation has been mixed on what is more efficacious. For example, the most recent Cochrane review in 2019 reported moderate certainty that there is no difference in cigarette cessation between abrupt or reduction cessation methods; however, a recent meta-analysis on cigarette cessation reported lower quit rates when NRT and reduction methods were used compared to NRT and abrupt cessation (35, 36). Giving individuals the autonomy to pick what works best for them in terms of cessation is an important feature of our intervention. There are many factors affecting quit rates, including self-efficacy levels, past quit attempts, and motivation that may be at play when participants decide whether to quit via reduction or abrupt methods (35), and these should be explored in future studies on SLT cessation. Because we heard from First Responders that autonomy in their cessation approach was important, we feel that it would be important to tailor cessation approaches to preferences in future trials to maximize efficacy.

Limitations of the current study include the small, underpowered sample and the homogeneity of our sample as most of the sample was male and white. This limits the generalizability of our findings; however, it is in line with national prevalence data on SLT use rates (37). The reliance on self-report cessation data (rather than verifying self-report cessation with cotinine testing) is also a limitation and may have inflated our actual quit rates. Strengths of the study include providing valuable data on the feasibility, acceptability, and preliminary efficacy of the first text-based SLT cessation intervention among First Responders, an at-risk group for poor health outcomes given the cancer risks associate with increased tobacco use and unique occupational exposures (38). Future research should test this intervention in a large-scale trial to determine efficacy.

## Data Availability

The raw data supporting the conclusions of this article will be made available by the authors without undue reservation.
